# Developing a framework to assess off-patent medicines policies in Latin America and the Caribbean

**DOI:** 10.1080/20523211.2026.2651399

**Published:** 2026-04-27

**Authors:** Jaime Espín, Santiago Palacio-Ciro, Ana Amaris, Pamela Gongora-Salazar, Veronika J. Wirtz

**Affiliations:** a Andalusian School of Public Health, Escuela Andaluza de Salud Pública (EASP), Granada, Spain; b CIBER en Epidemiología y Salud Pública (CIBERESP), Spain / CIBER of Epidemiology and Public Health (CIBERESP), Madrid, Spain; c Instituto de Investigación Biosanitaria ibs, Granada, Spain; d Centre for Health Economics, University of York, York, UK; e Consultant, Washington DC, USA; f Health, Nutrition and Population Division, Inter-American Development Bank, Washington, DC, USA; g Department of Global Health, Boston University School of Public Health, Boston, MA, USA

**Keywords:** Off-patent medicines, Generics, Biosimilars, Pharmaceutical policies, Latin America and the Caribbean, Scoping review

## Abstract

**Background:**

Countries in Latin America and the Caribbean (LAC) often lack a systematic approach to assessing the maturity and effectiveness of their policies for promoting off-patent (generic and biosimilar) medicines. Currently, no comprehensive, region-specific methodology exists that encompasses both generics and biosimilars. The objective of this paper is to develop a framework for assessing the performance of off-patent medicines policies that LAC countries can use to identify opportunities to improve access to these medicines in their territories.

**Methods:**

Through a scoping review of over 300 studies, this study identifies key supply- and demand-side policy interventions to promote generics and biosimilars, along with corresponding outcome indicators. This informs a logic-model-based framework, where supply-side policies are organized according to the pharmaceutical value chain, and demand-side policies are based on the target population.

**Results:**

The framework maps key policy actions, such as regulation, pricing, and substitution, to expected outcomes, such as affordability, uptake, and trust. The review highlights major regional gaps, including insufficient biosimilar policies, limited empirical evaluations, and the unique duality between public and private healthcare sectors in LAC. The existing literature gives greater attention to demand-side policies as potential factors driving higher market participation in generic and biosimilar medicines. Within this component, the emphasis is on factors aimed at education and awareness initiatives for users (41.6%). Supply-side policies are discussed less frequently, with price regulation (28.9%) most cited. By contrast, limited attention is given to other key factors such as horizon scanning, and mark-up regulation.

**Conclusion:**

The proposed framework serves as a diagnostic, analytical, and benchmarking instrument for policymakers and academia. It facilitates structured assessment, supports policy design, and fosters regional learning. Piloting and further empirical research are recommended to refine and validate the framework using a specific tool designed for this purpose.

## 1. Background

Pharmaceutical spending is one of the main drivers of health expenditure worldwide, often accounting for a substantial share of household out-of-pocket (OOP) costs, especially among low-income populations (WHO, 2023). Since OOP spending is one of the most inequitable ways to finance health care, it significantly slows economic development and exacerbates poverty. In many developing countries, high prices of medicines contribute to limited access to essential treatments and financial hardship. In countries like the Philippines, medicines represent 75% of OOP health spending among the poorest households, compared to 58% among the wealthiest (Bredenkamp & Buisman, 2016). In Mongolia, this figure rises to over 82% among the lowest income quintile (WHO, 2022). Latin America and the Caribbean (LAC) are not an exception to these challenges, as medicines account for a significant portion of both government health spending and household expenses. In Chile, for instance, medicines constitute 40% of household OOP spending, and direct household payments comprise 32% of total health expenditure (Atal et al., 2023). These figures underscore a persistent regional challenge: ensuring equitable, affordable access to medicines in health systems where financial protection mechanisms remain limited.

Off-patent medicines, particularly generics and biosimilars, offer an effective strategy to reduce costs, expand access to treatment, and enhance the sustainability of healthcare systems (Cameron et al., 2012; Morin et al., 2021). Generics contain the same active ingredients in the same strength and pharmaceutical form as the reference medicine, and their bioequivalence must be demonstrated through appropriate studies (WHO, 2006). Generic medicines can be sold either under a brand name or without one. Biosimilars are biological products that are shown to be highly similar in terms of their quality, safety and efficacy to an already licensed reference product (WHO, 2022). Evidence shows that substituting brand-name medicines with generic equivalents can generate substantial savings (IADB, 2023). Biosimilars, while more complex and costly to produce, also hold significant potential for expanding access to high-cost biologics (Aguilera et al., 2023). However, despite decades of global and regional policy guidance, including efforts led by the World Health Organization (WHO) / Pan American Health Organization (PAHO), the uptake of generics – and especially biosimilars – across LAC remains suboptimal. Leopold et al. (2023) highlighted that a key lesson for LAC from Europe is the potential benefits of adopting a tailored mix of pricing, reimbursement, and demand-side policies, especially those promoting the use of generics and biosimilars.

The barriers are multifaceted. Regulatory fragmentation, limited enforcement of bioequivalence, and resource constraints hinder the approval and oversight of generics and biosimilars in many countries (de la Cruz et al., 2017; PAHO, 2023). Market concentration, weak competition, and legal delays restrict timely market entry and limit the number of competitors, particularly in the biosimilar space (Leon et al., 2024). Meanwhile, public perception and trust challenges persist, with both prescribers and patients in some contexts continuing to prefer branded options (Aguilera et al., 2023). The lack of incentives to market, pricing regulations and harmonized regulatory standards compromises the availability and affordability of these medicines, especially in underserved areas (Leon et al., 2024).

For generic medicines, one major issue is the variation in excipients compared to the originator product. These differences can influence drug stability, absorption, and tolerability, potentially impacting therapeutic outcomes. The challenge is even greater for complex formulations such as modified-release tablets, inhalers, and injectables, where differences in delivery mechanisms and site-specific action complicate the demonstration of therapeutic equivalence (Blume et al., 2021). To mitigate some of these challenges, regulatory agencies have introduced biowaivers, which allow for in vitro testing instead of in vivo bioequivalence studies for certain drugs, particularly those with high solubility and permeability (WHO, 2006). This approach significantly reduces development costs and is especially beneficial for low- and middle-income countries, where conducting full-scale clinical trials may be financially and logistically prohibitive. Biosimilars, however, present a distinct set of challenges. Unlike generics, biosimilars are derived from living organisms and require extensive comparative clinical trials to demonstrate that there are no clinically meaningful differences from the reference biologic in terms of safety, purity, and potency (WHO, 2022). These trials must also assess immunogenicity and efficacy in sensitive populations, making the development process both expensive and time-consuming. To address these barriers, global harmonization efforts are underway. Regulatory frameworks aim to streamline approval processes and reduce duplication in regulatory requirements, while maintaining rigorous standards for safety and efficacy (PAHO, 2022). These initiatives are crucial for enabling broader access to biosimilars and generics, especially in resource-limited settings.

Hence, addressing these challenges requires more than policy recommendations; it requires tools that allow governments and institutions to diagnose existing barriers, prioritize interventions, and monitor progress. However, countries in LAC often lack a region-specific systematic approach to assessing the maturity and effectiveness of their policies for promoting generics and biosimilars. While frameworks exist to evaluate the performance of generic medicines (e.g., Kanavos, 2014), none provides a comprehensive, regionally adapted methodology that includes both generics and biosimilars.

This study aims to fill that gap by developing an assessment framework (hereafter, the Framework) designed to support LAC countries in assessing their policy landscape for generic and biosimilar medicines and identifying opportunities to strengthen it.

## 2. Methods

A scoping literature review was conducted to (i) investigate the policies used to promote generics and biosimilars, and (ii) identify the main indicators to measure the impact of policies promoting these medicines. For the purpose of this paper, the term ‘off-patent medicines’ refers exclusively to generic and biosimilar medicines. The ultimate objective of this review is to help develop a framework for assessing generic and biosimilar policies in LAC. Promoting these medicines involves not only expanding their market share but also ensuring the availability of quality-assured products and fostering users’ trust in their quality, among other considerations. This review, therefore, focused on policies across the pharmaceutical value chain, including policies that influence the development, approval, market entry, pricing, reimbursement, prescribing, dispensing, and use of generics and biosimilars.

### 2.1. Data sources and search strategy

A systematic search was performed across a comprehensive range of bibliographic databases and websites, including MedLine (PubMed), Literatura Latino-Americana e do Caribe em Ciências da Saúde (LILACS), Scopus, Web of Science, Embase, Econlit, Cochrane Effective Practice and Organisation of Care Group, National Health Service Economic Evaluation Database (NHS EED), The National Bureau of Economic Research (NBER), World Health Organization, InterAmerican Development Bank, European Commission, Organization for Economic Cooperation and Development, and the World Bank. To identify gray literature, Google Scholar and TripDatabase were also searched. Additionally, the websites of national regulatory agencies and ministries of health for countries in LAC were reviewed for relevant off-patent medicine policies and legal documents.

The search strategy was developed using Boolean operators and thematic keywords with guidance from a librarian across four conceptual areas: (1) Generic and biosimilar medical products: keywords included generic drugs, biosimilars, off-patent medicines, non-proprietary drugs, multi-source products, biopharmaceuticals, and non-innovators; (2) Policies and Interventions: keywords included health policy, pharmaceutical policy, regulatory policies, education campaigns, and procurement policies; (3) Promotion and uptake: keywords included promotion, adoption, uptake, utilization, acceptance, market penetration, and policy impact. (4) Impact measurement: keywords included indicators, policy evaluation, outcomes assessment, economic impact, health outcomes, and market share.

We started searching for titles that included the first block of keywords and then combined that block with the other three using every possible iteration – eight sets of combinations of keywords in total. Example search strings included combinations such as: (generic drugs OR biosimilars OR off-patent medicines) AND (promotion OR adoption OR utilization OR market penetration) AND (indicators OR policy evaluation OR economic impact OR health outcomes).

Searches were conducted by two researchers (AA and SPC) and documented for each database, including the complete search string, date of search, database used, number of hits, and number of relevant articles identified. The research results were reviewed by AA, SPC, VW and JE. More on the search strategies can be found in Supplemental Material A.

### 2.2. Inclusion/exclusion criteria

In this review, the following inclusion criteria were chosen: publications addressing the research objectives, studies discussing indicators or frameworks for evaluating the success of policies aimed at promoting generic or biosimilar medicines, research on the promotion, adoption, or utilization of generic medicines or biosimilars with policy- or market-driven factors, empirical studies, systematic reviews, meta-analyses, or case studies evaluating the impact of policies on economic, health, or market-related outcomes. Searches covered studies published between January 2000 and March 2025 in English, French, Spanish, Portuguese, or German. The review included global literature without geographic restrictions.

On the other hand, publications with insufficient methodological detail (e.g. posters, commentaries, opinion pieces, editorials) were excluded, as were publications predating 2000. For the purpose of this study, *medicinal products* refer to pharmaceutical products used for therapeutic treatment, including both chemical and biological medicines. Preventive health technologies such as vaccines, and non-pharmaceutical products such as medical devices, were excluded to maintain focus on policies governing off-patent medicines.

### 2.3. Data management and analysis

All search results were managed using Covidence (2024) software to facilitate screening and data extraction. All duplicates were eliminated before data extraction. Two of the researchers (AA and SPC) reviewed each publication. Those identified as relevant were recorded in a spreadsheet that documented key details, including authors, title, publication year, abstract, and journal. A review matrix in Excel was created for included studies, capturing essential data elements such as the study's focus, geographic area, methodology, findings, and relevance to the research questions. All titles and abstracts were screened independently by two researchers (AA and SPC). In cases of disagreement or doubt on study inclusion, a third and fourth researcher (VW, JE) reviewed the publication, and consensus was reached through discussion.

The authors extracted the factors that studies had identified as relevant to promoting the uptake of generic or biosimilar medicines from the Excel spreadsheet. These factors were arranged by supply-side and demand-side, and then mapped according to the stages of the pharmaceutical value chain (WHO, 2021). A visual representation of the Framework was developed in line with a logic model, where the activities enacted through policies are arranged on the left side of the diagram and the outcomes of the policies on the right side of the diagram (see Figure 3).

To support the conceptual development of this framework, we selected literature reviews and descriptive studies on generic and biosimilar policies to illustrate each of the policies identified. From the broader pool of studies, we prioritized those that aligned more closely with our research objective of developing an assessment framework to support LAC countries in evaluating and strengthening their policy landscape for generics and biosimilars. Articles were further assessed based on the approach used to discuss key policy areas, the strength of their methodologies, and their potential to inform the structure and content of our proposed framework. We then applied a color-coded classification system to organize the retrieved articles by relevance.

### 2.4. Reporting and framework development

The review followed the Preferred Reporting Items for Systematic Reviews and Meta-Analyses (PRISMA) guidelines and the extension for Scoping Reviews Checklist to ensure transparency and comprehensiveness (Page et al., 2021; Tricco et al., 2018). The findings were synthesized to inform the development of the Framework for assessing generic and biosimilar policies in LAC, highlighting gaps and providing recommendations for policy and research.

Based on the findings from the literature, we developed a framework of policies promoting generic and biosimilar medicines (referred to as the Framework) in the form of a logic model to illustrate how different policies can promote the adoption and use of these off-patent medicines. For the purposes of this work, ‘policies’ are defined broadly as governmental, regulatory, and institutional rules, standards, and actions aimed at influencing the availability, pricing, market entry, prescribing, dispensing, and use of generics and biosimilars across the pharmaceutical value chain.

In building the Framework, the evidence extracted from each included article was first mapped against the different components of the pharmaceutical value chain, and articles were sorted accordingly (Supplemental Material B). Identified policies were then grouped by supply-side and demand-side levers, maintaining the value-chain logic. In a final step, we extracted and classified the outcomes reported in the literature (e.g. price, availability, user acceptance) and aligned them with the corresponding policy pathways. This iterative process was conducted jointly by JE, VW, and PGS, through several discussion sessions, which ensured consensus in the interpretation, classification, and scope of the Framework.

## 3. Results

### 3.1. Search results

Using databases and registered websites, a total of 715 records were screened after 461 duplicate records were removed. Publication titles were reviewed. Out of them, 404 were excluded because their title or abstract was found to be not relevant. For the remaining 311 records, the full text was retrieved for 292 records. Nineteen full-text publications were excluded because either they were not relevant, the methodology was not sufficiently described, they were not available, or the publication focused on the consequences of the policies promoting biosimilars or generic medicines rather than the policies themselves.

**Figure 1.**
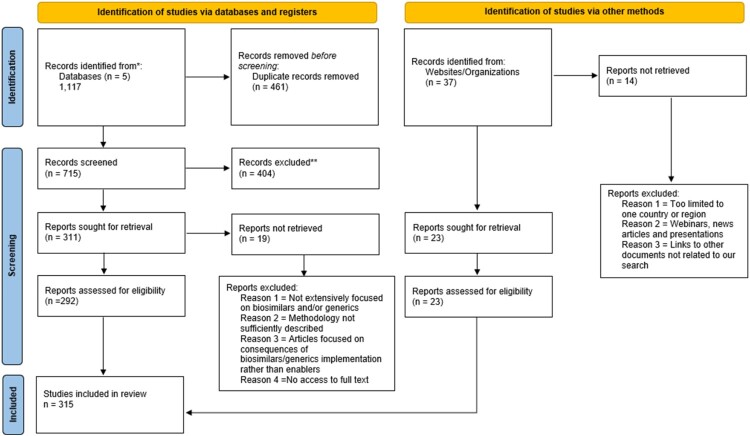
PRISMA flow diagram. Source: Own elaboration based on (Page et al., 2021).

Thirty-seven records were identified through the review of the websites of five large international organizations, such as the WHO, the PAHO, and the Organization for Economic Cooperation and Development (OECD). Of these, 23 were found relevant. Figure 1 presents the flowchart of the literature review, as outlined in the PRISMA guidelines.

**Figure 2.**
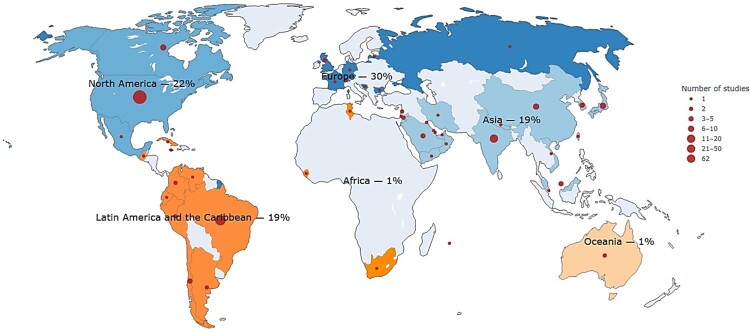
Geographic distribution of included studies.

Out of the 315 articles included in the review, 203 (64%) focused on generic medicines, 88 (28%) on biosimilars, and 24 (8%) addressed both. 274 (87%) were published in English, 22 (7%) in Portuguese, and 19 (6%) in Spanish. Regarding geographic location, 96 (30%) of these studies are from European countries, 61 (19%) from LAC countries (mainly Brazil, which represents 35 studies or 11% of the total), 62 (20%) from the US, and 38 (12%) from studies including countries from all around the world. The remaining proportion corresponds to a mix of countries or other regions, such as Western Pacific and Middle East and North Africa (MENA). Figure 2 further illustrates the geographic distribution of the studies.

There are five literature reviews focused on generic policies (Dylst et al., 2014; Kaló et al., 2015; Kanavos, 2014; Kaplan et al., 2012; King & Kanavos, 2002) and one on biosimilar medicines policies (Machado et al., 2024). Most of them focus on European countries, except for Kaplan et al. (2012), with an overall focus on low- and middle-income countries. The studies provide a detailed description of policies that have been used to promote generics and biosimilars. While 315 studies met the inclusion criteria, a subset of 33 publications was selected to illustrate the Framework. These studies were chosen based on methodological quality, policy relevance, and representativeness across supply- and demand-side policy domains, as detailed in Supplemental Material B. Most of the 33 selected studies analyzed policies at the system or market level rather than by therapeutic class. A few studies referenced specific product groups, such as oncology biologics, primarily to illustrate policy application within those contexts. Supplemental Material B provides a critical synthesis of the 33 selected studies, summarizing their scope, methods, and key findings. This synthesis served as the empirical foundation for structuring the Framework and mapping policy interventions to expected outcomes. Supplemental Material C presents a graphical overview of the literature gaps related to factors influencing the promotion, adoption, and utilization of off-patent medicines. This literature places greater emphasis on demand-side measures, particularly education and awareness campaigns targeting medicine users (41.6%) and incentives for prescribers and dispensers (40.3%). In comparison, supply-side measures are discussed less frequently, with price regulation (28.9%) and reference systems (17.5%) receiving the most attention. Evidence on the role of horizon scanning, HTA, or budget impact analyses, and mark-up regulation is limited, with each appearing in fewer than 6% of the reviewed studies. Aspects related to market authorization are more unevenly addressed, with most attention given to bioequivalence testing (12.4%) and market exclusivity periods (9.2%), while the Bolar rule and parallel imports are rarely mentioned (each 0.3%).

### 3.2. Proposed framework

**Figure 3.**
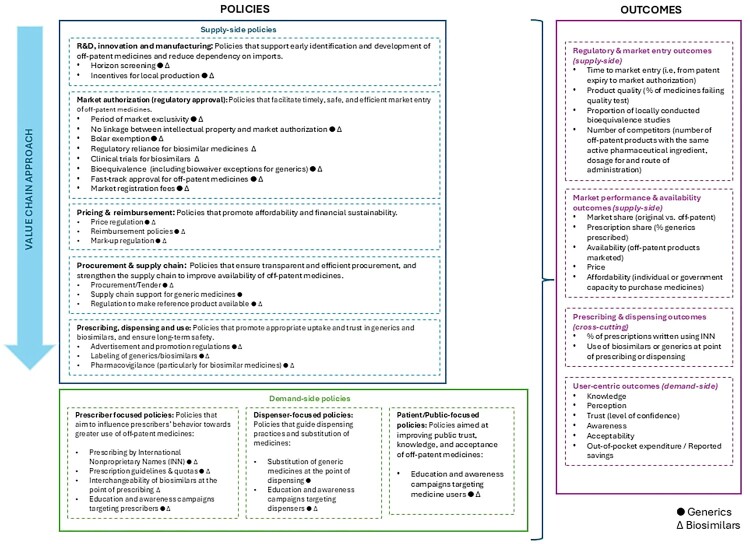
Framework of policies and expected outcomes for promoting off-patent medicines (generics and biosimilars).

On the left-hand side of the Framework (Figure 3), policies were arranged and grouped into two broad categories: supply-side and demand-side. Supply-side policies were organized according to the pharmaceutical value chain, comprising five domains: (i) research and development, innovation, and manufacturing; (ii) market authorization; (iii) pricing and reimbursement; (iv) procurement and supply chain management; and (v) prescribing, dispensing, and use. Demand-side policies, aiming at promoting further use of generics and/or biosimilars, were structured based on the target population: prescribers, dispensers, patients and the general public. On the right-hand side, expected outcomes are presented, linking each policy group to its intended effects on market uptake and use of off-patent medicines. Indicators to measure the intended effect include time to market entry for off-patented medicines after patent expiry, the market share of patented medicines versus off-patented medicines, the share of generic medicines prescribed, and capacity of individuals or the government to purchase medicines. Example indicators to measure quality of off-patent products include among others the proportion of off-patented medicines failing the quality test.

The Framework is a simplification of reality, omitting the interplay between different activities and the extent to which they generate the expected outcomes. It is not the aim of the Framework to be explanatory in terms of the interactions between policies, because evidence does not provide sufficient information about how and in what order policies must interact to produce the desired outcomes.

### 3.3. Supply-side policies and their outcomes

Out of the 33 studies that describe at least one supply-side policy, 14 focus on LAC (Supplemental Material B). Of those, seven are regional studies (Cox, 2013; Leon et al., 2024; Lizarraga & Mysler, 2019; PAHO, 2022; Rosselli et al., 2023; Teran et al., 2022; Vargas et al., 2022), five focus exclusively on Brazil (Bertoldi et al., 2019; Da Fonseca, 2014; Dias & Romano-Lieber, 2006; Pimenta Menteiro et al., 2019; Scheinberg et al., 2018), two on Chile (Atal et al., 2023; Stojanova et al., 2020), and one on the Caribbean (Cox, 2013).

In terms of supply-side policies, our review identified two policies related to R&D, innovation, and manufacturing; eight on market authorization; three on pricing and reimbursement; three on procurement and supply chain; and three on prescribing, dispensing, and use. Policies in the R&D, innovation, and manufacturing domains generally aim to promote the early identification and development of off-patent medicines and reduce reliance on imports. In LAC, incentives for local production have been particularly emphasized in Brazil, where initiatives highlight efforts to strengthen national capacity (Pimenta Menteiro et al., 2019; Scheinberg et al., 2018), while regional strategies have also been reported (Teran et al., 2022).

Market authorization policies focus on facilitating the timely, safe, and efficient entry of off-patent medicines into the market. Examples include clinical trial requirements for biosimilars and bioequivalence testing to ensure product quality. Policies such as the period of market exclusivity, the Bolar exemption, and fast-track approval for off-patent medicines are intended to shorten the time to market. Hence, indicators measure the effect of these policies, including time to entry and quality testing results. In LAC, studies describe both exclusivity periods and the application of the Bolar exemption (Vargas et al., 2022), while the role of regulatory reliance has been emphasized as a strategy to expedite biosimilar approvals (PAHO, 2022). Moreover, evidence from Brazil and Chile suggests the dual role of bioequivalence – while it helps increase prescriber and patient trust, it has also led to increased manufacturing costs (Atal et al., 2023; Da Fonseca, 2014).

Pricing and reimbursement policies aim to promote affordability and financial sustainability by encouraging the use of cost-effective alternatives. For instance, reimbursement policies that favor generics and biosimilars, such as reference pricing systems or tiered copayment structures, can incentivize their selection over higher-cost originators. The most relevant LAC example is the ‘Farmacia Popular’ program in Brazil, where the government introduced copayments in private pharmacies to nudge and encourage the use of lower-priced products (Bertoldi et al., 2019). Mark-up regulation is another area where LAC experience is distinct: while mark-ups are not formally regulated in most countries, they represent a significant cost burden to consumers (Leon et al., 2024).

Procurement and supply chain policies aim to ensure transparent, competitive, and efficient procurement processes, while strengthening distribution systems to enhance the availability of off-patent medicines, particularly in underserved areas. In LAC, studies provide insight into procurement practices (Rosselli et al., 2023) and describe supply chain challenges for biosimilars in Brazil (Scheinberg et al., 2018).

### 3.4. Demand-side policies and their outcomes

Regarding demand-side policies, we identified 22 studies describing at least one policy targeting prescribers, dispensers, or consumers (Supplemental Material B). Eight of them focus on LAC: three regional studies (Aguilera et al., 2023; PAHO, 2022; Teran et al., 2022), two on Chile (Atal et al., 2023; Stojanova et al., 2020), one on Argentina (Tobar, 2008), one on Brazil (Bertoldi et al., 2019), and one on Colombia (Torres Serna et al., 2018).

Policies related to prescribing, dispensing, and use are designed to promote the appropriate uptake of generics and biosimilars, build trust in these products, and ensure their long-term safety. For example, clear and consistent labelling of generics and biosimilars supports informed prescribing. In LAC, labelling requirements have been highlighted in Brazil and Chile, where distinct packaging of generics was found to enhance recognition and confidence among patients (Da Fonseca, 2014; Dias & Romano-Lieber, 2006; Stojanova et al., 2020). Outcome indicators to assess performance and availability focus on measuring the market share of off-patent medicines, prescription share, and the number of competitors of off-patent products. This category also includes outcome indicators to assess affordability, focusing on the individual or government capacity to purchase off-patent medicines. For individual affordability, this includes comparing prices to the minimum salary, for instance.

Prescription policies,– such as the use of quotas to promote biosimilar medicines,– have received considerable attention in the European literature; however, no publication from LAC has provided a detailed description of how such policies are implemented in the region. One cornerstone demand-side policy is substitution, which is widely used in Europe, either on a mandatory or voluntary basis. In LAC, the experience is more diverse: in Chile, substitution requires patient consent, resulting in lower generic uptake (Atal et al., 2023; Stojanova et al., 2020), while in Brazil, pharmacists are permitted to substitute without patient request, facilitating broader use (Bertoldi et al., 2019).

Similarly, policies aimed at increasing prescribers’, dispensers’, and patients’ knowledge, beliefs, and attitudes toward off-patent medicines have been highlighted in the literature. Regional studies emphasize awareness campaigns and guidelines (Aguilera et al., 2023; PAHO, 2022), while evidence from Colombia illustrates the importance of public information (Torres Serna et al., 2018). Notably, two publications underscore the urgent need to clarify interchangeability guidelines for biosimilars in the region (PAHO, 2022; Teran et al., 2022).

A glossary describing each policy included in the Framework is provided in Supplemental Material D.

## 4. Discussion

This study introduces a logic model framework for assessing the maturity and effectiveness of policies promoting off-patent medicines in the LAC region. Previous efforts to analyze off-patent medicine promotion have generally focused either on policies (Kaló et al., 2015; Vogler et al., 2017) or on outcomes (Kanavos, 2014). This framework combines policies and expected outcome measures into a single logic model, where policies are regarded as interventions and activities, and the anticipated effects of these policies are considered outcomes. The Framework responds to the critical need for structured tools that can guide countries in evaluating their pharmaceutical policy environments and identifying opportunities for improvement. By organizing policy interventions into supply-side and demand-side categories and linking them to measurable outcomes, the Framework offers a practical and comprehensive approach for policy assessment. Particularly, by aligning policies with the pharmaceutical value chain, it facilitates the identification of key policy areas across the medicine life cycle. It helps pinpoint the relevant stakeholders needed for successful implementation.

In developing the Framework, we emphasized evidence from LAC and highlighted policy components that resonate with the region’s health system structure. The Framework developed in this study aligns with broader goals of enhancing health system efficiency and financial protection by promoting the appropriate use of lower-cost, quality-assured medicines.

The Framework can also serve as a foundation for a standardized assessment tool, usable for cross-country comparisons, monitoring over time, and informing regional policy dialogues in LAC. Its adaptability allows for tailoring to national contexts while maintaining a consistent structure for evaluation. It can serve as a diagnostic tool to assess the current status of policies and identify gaps, an analytical tool to evaluate the effectiveness of interventions over time, and a comparative tool to benchmark progress across countries or subregions.

All elements in the Framework are referenced in the literature and implemented in several countries. However, the strength of evidence for their effect varies. Indeed, we found only a few studies that rigorously evaluate the impact of the policies. While mandatory substitution at dispensing shows strong evidence of effectiveness in increasing generic use (Andersson et al., 2008), other policies lack evidence, such as the regulation of markups (Ball, 2014; Leon et al., 2024). This does not imply that the latter are less effective.

It is noteworthy that most of the literature on off-patent medicines in LAC overlooks the large fragmentation of health systems, where the public sector integrates financing, procurement, prescribing, and dispensing within one institution, while the private sector largely relies on OOP spending. Generally, patients whose care is financed through public funds do not pay or only pay a small fee or a proportion of the price (Bossert et al., 2014; PAHO, 2014). Contrastingly, the private sector consists of thousands of private healthcare providers and retail pharmacies; patients pay OOP for the use of each service. As a result, governments must decide whether policies promoting off-patent medicines are intended to target the public or the private sector, since both operate very differently in terms of financing, payments, and regulation. The fact that we did not find literature describing *reimbursement* policies of off-patent medicines in LAC except two studies (Bertoldi et al., 2019; Tobar, 2008) is due to the structure of the health systems where in the majority of cases medicines are paid either OOP at the retail pharmacy counter or by institutions such as Ministries of Health or social security through their tender process. Instead, policies such as those targeting the private sector through direct price regulation (e.g. markups), prescription (e.g. INN), or educational campaigns seem more relevant. This is particularly important since the public sector has generally implemented a series of policies, such as tender policies, prescription policies, and guidelines, resulting in a very high percentage of off-patent medicines being used.

The literature on biosimilar policies in LAC primarily focuses on regulatory maturity and manufacturing, rather than incentives, to encourage uptake in both the public and private sectors. Promoting the use of biosimilars in public institutions can be achieved through standard treatment guidelines, mandatory prescriber training, and prescription audits. Incentivizing biosimilar use in private hospitals, however, is challenging. For example, Lizarraga et al. (2019) highlighted the most important challenges for Latin America regarding biosimilars as ‘switching, substitution, naming, extrapolation of indications and pharmacovigilance’, and requesting that ‘biosimilarity should be granted through pre-clinical and clinical head-to-head studies comparing the biosimilar with the innovator product’. Nevertheless, if we compare with European countries or the US, for example, the inequities in access to therapy based on economic differences and the absence of harmonization of regulatory guidance should also be considered as relevant aspects for LAC countries.

Despite the breadth of literature reviewed, several gaps were identified that limit the explanatory power of the Framework. First, there is a lack of empirical studies evaluating the causal impact of specific policies on access, affordability, or health outcomes, particularly in LAC. We identified only a handful of studies that measure the impact of generic policies (Patel et al., 2021; Goldszmidt et al., 2019). Most focus on policy descriptions or theoretical benefits, with limited data on implementation fidelity or real-world effectiveness, which restricts the analysis of interactions and sequencing of interventions. Therefore, further empirical research is needed to validate the Framework, examine causal pathways, and refine indicators for measuring policy effectiveness. A second limitation is that the Framework does not provide information on the sequence and synergies of different policies to promote generic and biosimilar medicines. Literature suggests that effective strategies require synchronized approaches integrating regulatory reform, pricing, procurement, education, and stakeholder engagement. For instance, price regulation alone is unlikely to succeed without reimbursement incentives or public education campaigns. Similarly, the benefits of faster regulatory approval pathways can only be realized if complemented by effective post-market surveillance and quality assurance systems. Countries relying on piecemeal reforms may find such interventions insufficient to shift entrenched behaviors or alter market dynamics. Third, there are significant content gaps in the literature, such as those related to biosimilars, which remain underrepresented compared to generics, reflecting both their relatively recent introduction and the complexity of their regulation and market dynamics. This imbalance may skew the Framework toward generic-focused policies, underscoring the need for further research on biosimilar-specific challenges and enablers in the region. Other policies, such as markup regulation and Bolar rules, are also underrepresented. Consequently, the application of the Framework to biosimilars may require supplementary country-specific information or expert judgement until more evidence becomes available. Fourth, only 18% of the reviewed studies originated from LAC, with most evidence drawn from European and North American experiences. While these provide valuable lessons, they may not fully reflect the institutional and market dynamics of the region. This imbalance reinforces the importance of validating and refining the Framework through country-specific applications and stakeholder consultations within LAC. Lastly, although the Framework encompasses a broad range of outcome indicators, data availability and quality vary significantly across countries. This may limit the feasibility of applying the Framework uniformly without additional investment in data systems and capacity building.

To move from a conceptual framework to a practical assessment tool, several steps are needed. First, the Framework should be piloted in a diverse set of LAC countries to test its usability, relevance, and adaptability. This process should include stakeholder consultations to refine indicators, ensure contextual appropriateness, and identify data sources. Second, efforts should be made to harmonize definitions and metrics across countries to enable meaningful comparisons and regional benchmarking.

Future research should focus on generating empirical evidence on the effectiveness of specific policy interventions in LAC, particularly in underrepresented areas such as biosimilars, markup policies and prescriber preferences. Mixed-methods studies combining quantitative indicators with qualitative insights from stakeholders could provide a richer understanding of policy dynamics and inform iterative improvements to the Framework.

## 5. Conclusion

This study proposes a logic model framework to evaluate policies promoting generics and biosimilars in LAC. It provides a promising step toward more systematic, evidence-informed policy evaluation in the pharmaceutical sector. By linking supply- and demand-side policies to measurable outcomes and contextualizing them within the LAC health system structure, it offers countries a structured approach to assess their pharmaceutical policies. Supporting countries in identifying strengths, gaps, and opportunities can contribute to more equitable and sustainable access to essential medicines across the region. Continued empirical research and piloting in diverse contexts will be key to refining the Framework and ensuring its value as a practical tool for policymakers.

## Supplementary Material

Supplemental Material
