# Supplementary material for: Developing a framework to assess off-patent medicines policies in Latin America and the Caribbean
Source: J Pharm Policy Pract. 2026 Apr 27;19(1):2651399. doi: 10.1080/20523211.2026.2651399 (PMC13123092; doi:10.1080/20523211.2026.2651399)
Supplement: Supplemental Material [file JPPP_A_2651399_SM8643.docx]

**Supplementary Material**

**Developing a framework to assess off-patent medicines policies in Latin America and the Caribbean**

1. **SEARCH STRATEGIES**

**Scopus**

**Filters applied:** Date: 2000-2025. Languages: English, French, German, Spanish, Portuguese. Document type: Article, review, book chapter, conference paper, book. Excluding medical devices and vaccines (AND NOT operator).

The (*) operator makes a big difference in this search database. For example, for the first string, the number of hits without the operator was half that with its inclusion.

**Search strings**

Generics and policies

| Generic and Biosimilar Medical Products | (AND) Policies and Interventions |
| --- | --- |
| ("generic drug*" OR biosimilar* OR "off-patent medicine*" OR "non-proprietary drug*" OR biopharmaceutical* OR "generic medicine*") | (policy OR regulation OR law OR strategy) |

Generics and promotion/uptake

| Generic and Biosimilar Medical Products | (AND) Promotion and Uptake |
| --- | --- |
| ("generic drug*" OR biosimilar* OR "off-patent medicine*" OR "non-proprietary drug*" OR biopharmaceutical* OR "generic medicine*") | (promotion OR adoption OR uptake OR utilization OR “market penetration”) |

Generics and impact measurement

| Generic and Biosimilar Medical Products | (AND) Impact Measurement |
| --- | --- |
| ("generic drug*" OR biosimilar* OR "off-patent medicine*" OR "non-proprietary drug*" OR biopharmaceutical* OR "generic medicine*") | (indicators OR “policy evaluation” OR “outcomes assessment” OR “health outcomes”) |

Generic and Biosimilar Medical Products, Policies and Interventions, and Promotion and Uptake

| Generic and Biosimilar Medical Products | (AND) Policies and Interventions | (AND) Promotion and Uptake |
| --- | --- | --- |
| ("generic drug*" OR biosimilar* OR "off-patent medicine*" OR "non-proprietary drug*" OR biopharmaceutical* OR "generic medicine*") | (policy OR regulation OR law OR strategy) | (promotion OR adoption OR uptake OR utilization OR “market penetration”) |

Generic and Biosimilar Medical Products, Policies and Interventions, and Impact Measurement

| Generic and Biosimilar Medical Products | (AND) Policies and Interventions | (AND) Impact Measurement |
| --- | --- | --- |
| ("generic drug*" OR biosimilar* OR "off-patent medicine*" OR "non-proprietary drug*" OR biopharmaceutical* OR "generic medicine*") | (policy OR regulation OR law OR strategy) | (indicators OR “policy evaluation” OR “outcomes assessment” OR “health outcomes”) |

Generic and Biosimilar Medical Products, Promotion and Uptake, and Impact Measurement

| Generic and Biosimilar Medical Products | (AND) Promotion and Uptake | (AND) Impact Measurement |
| --- | --- | --- |
| ("generic drug*" OR biosimilar* OR "off-patent medicine*" OR "non-proprietary drug*" OR biopharmaceutical* OR "generic medicine*") | (promotion OR adoption OR uptake OR utilization OR “market penetration”) | (indicators OR “policy evaluation” OR “outcomes assessment” OR “health outcomes”) |

Generic and Biosimilar Medical Products, Policies and Interventions, Promotion and Uptake, and Impact Measurement

| Generic and Biosimilar Medical Products | (AND) Policies and Interventions | (AND) Promotion and Uptake | (AND) Impact Measurement |
| --- | --- | --- | --- |
| ("generic drug*" OR biosimilar* OR "off-patent medicine*" OR "non-proprietary drug*" OR biopharmaceutical* OR "generic medicine*") | (policy OR regulation OR law OR strategy) | (promotion OR adoption OR uptake OR utilization OR “market penetration”) | (indicators OR “policy evaluation” OR “outcomes assessment” OR “health outcomes”) |

**Web of Science**

The (*) operator makes a big difference in this search database. For example, for the first string, the number of hits without the operator was half of that with its inclusion.

**Filters applied:** Date: 2000-2025. Languages: English, French, German, Spanish, Portuguese. Document type: Article or review article, proceeding paper, book chapters, or early access. Excluding vaccines and medical devices -Not (vaccine* OR medical device*).

Generics and policies

| Generic and Biosimilar Medical Products | (AND) Policies and Interventions |
| --- | --- |
| (generic drug* OR biosimilar* OR off-patent medicine* OR "non-proprietary drug*" OR biopharmaceutical OR generic medicine) | (policy OR regulation OR law OR strategy) |

Generics and promotion/uptake

| Generic and Biosimilar Medical Products | (AND) Promotion and Uptake |
| --- | --- |
| (generic drug* OR biosimilar* OR off-patent medicine* OR non-proprietary drug* OR biopharmaceutical OR generic medicine) | (promotion OR adoption OR uptake OR utilization OR market penetration) |

Generics and impact measurement

| Generic and Biosimilar Medical Products | (AND) Impact Measurement |
| --- | --- |
| (generic drug* OR biosimilar* OR off-patent medicine* OR non-proprietary drug* OR biopharmaceutical OR generic medicine) | (indicators OR policy evaluation OR outcomes assessment OR health outcome*) |

Generic and Biosimilar Medical Products, Policies and Interventions, and Promotion and Uptake

| Generic and Biosimilar Medical Products | (AND) Policies and Interventions | (AND) Promotion and Uptake |
| --- | --- | --- |
| (generic drug* OR biosimilar* OR off-patent medicine* OR non-proprietary drug* OR biopharmaceutical OR generic medicine) | (policy OR regulation OR law OR strategy) | (promotion OR adoption OR uptake OR utilization OR market penetration) |

Generic and Biosimilar Medical Products, Policies and Interventions, and Impact Measurement

| Generic and Biosimilar Medical Products | (AND) Policies and Interventions | (AND) Impact Measurement |
| --- | --- | --- |
| (generic drug* OR biosimilar* OR off-patent medicine* OR non-proprietary drug* OR biopharmaceutical OR generic medicine) | (policy OR regulation OR law OR strategy) | (indicators OR policy evaluation OR outcomes assessment OR health outcome*) |

Generic and Biosimilar Medical Products, Promotion and Uptake, and Impact Measurement

| Generic and Biosimilar Medical Products | (AND) Promotion and Uptake | (AND) Impact Measurement |
| --- | --- | --- |
| (generic drug* OR biosimilar* OR off-patent medicine* OR non-proprietary drug* OR biopharmaceutical OR generic medicine) | (promotion OR adoption OR uptake OR utilization OR market penetration) | (indicators OR policy evaluation OR outcomes assessment OR health outcome*) |

Generic and Biosimilar Medical Products, Policies and Interventions, Promotion and Uptake, and Impact Measurement

| Generic and Biosimilar Medical Products | (AND) Policies and Interventions | (AND) Promotion and Uptake | (AND) Impact Measurement |
| --- | --- | --- | --- |
| (generic drug* OR biosimilar* OR off-patent medicine* OR non-proprietary drug* OR biopharmaceutical OR generic medicine) | (policy OR regulation OR law OR strategy) | (promotion OR adoption OR uptake OR utilization OR market penetration) | (indicators OR policy evaluation OR outcomes assessment OR health outcome*) |

**Econlit**

**Filters applied:** Date: 2000-2025. Languages: English, French, German, Spanish, Portuguese. Document type: Article, review, book chapter, conference paper, book. Excluding medical devices and vaccines (AND NOT operator).

**Search strings**

Generics and policies

| Generic and Biosimilar Medical Products | (AND) Policies and Interventions |
| --- | --- |
| ("generic drug*" OR biosimilar* OR "off-patent medicine*" OR "non-proprietary drug*" OR biopharmaceutical* OR "generic medicine*") | (policy OR regulation OR law OR strategy) |

Generic and promotion/uptake

| Generic and Biosimilar Medical Products | (AND) Promotion and Uptake |
| --- | --- |
| ("generic drug*" OR biosimilar* OR "off-patent medicine*" OR "non-proprietary drug*" OR biopharmaceutical* OR "generic medicine*") | (promotion OR adoption OR uptake OR utilization OR “market penetration”) |

Generics and impact measurement

| Generic and Biosimilar Medical Products | (AND) Impact Measurement |
| --- | --- |
| ("generic drug*" OR biosimilar* OR "off-patent medicine*" OR "non-proprietary drug*" OR biopharmaceutical* OR "generic medicine*") | (indicators OR “policy evaluation” OR “outcomes assessment” OR “health outcomes”) |

Generic and Biosimilar Medical Products, Policies and Interventions, and Promotion and Uptake

| Generic and Biosimilar Medical Products | (AND) Policies and Interventions | (AND) Promotion and Uptake |
| --- | --- | --- |
| ("generic drug*" OR biosimilar* OR "off-patent medicine*" OR "non-proprietary drug*" OR biopharmaceutical* OR "generic medicine*") | (policy OR regulation OR law OR strategy) | (promotion OR adoption OR uptake OR utilization OR “market penetration”) |

**Google Scholar**

**Settings:** Date: 2000-2025. Languages: English, French, German, Spanish, Portuguese.

Search 1. Putting everything together

Off-patent medicine* OR drug* OR molecule* OR generic* OR non-innovator OR biosimilar* OR biopharmaceutical* OR bioequivalence OR substitution pharmaceutical* OR interchangeable generics OR multi-source drug OR interchangeability AND health polic* OR pharmaceutical polic* OR policy making AND access barriers OR access policies AND health care industry OR local production AND regulatory landscape AND entry biologics -vaccine*

Search 2. Policy

Off-patent medicine* OR drug* OR molecule* OR generic* OR non-innovator OR biosimilar* OR biopharmaceutical* OR bioequivalence OR substitution pharmaceutical* OR interchangeable generics OR multi-source drug OR interchangeability AND health policy OR pharmaceutical policy OR policy making OR access polic*

Date: from the 2000’s

Search 3. Regulation

Off-patent medicine* OR drug* OR molecule* OR generic* OR non-innovator OR biosimilar* OR biopharmaceutical* OR bioequivalence OR substitution pharmaceutical* OR interchangeable generics OR multi-source drug OR interchangeability AND access barriers AND regulatory landscape OR regulation OR entry biologics

Date: from the 2000’s

Search 4. Market composition

Off-patent medicine* OR drug* OR molecule* OR generic* OR non-innovator OR biosimilar* OR biopharmaceutical* OR bioequivalence OR substitution pharmaceutical* OR interchangeable generics OR multi-source drug OR interchangeability AND health care industry OR local production

1. **KEY FINDINGS FROM PUBLICATIONS**

| **Factors** | | **Papers of reference** | **Region** | **Countries (if applicable)** | **Key findings** |  |
| --- | --- | --- | --- | --- | --- | --- |
| **Supply-side policies** | | | | | |  |
| **R&D, innovation and manufacturing** | | |  |  |  |  |
| **1** | **Horizon screening** | Moorkens et al. (2021) | Europe | Belgium | Horizon scanning support to prepare in advance for the patent expiry and entering of biosimilar into the market. This allows working towards alignment of stakeholders and taking into consideration new contracts. The future entry of products and the length of the contract should be determined accordingly. |  |
| **2** | **Incentives for local production** | Scheinberg et al. (2018) | LAC | Brazil | Emphasis on partnership programs between national government-owned biosimilar companies and private technology holders have been implemented, aiming at knowledge sharing, capacity-building and technological transfer. |  |
|  |  | Pimenta & Monteiro (2019) | LAC | Brazil | Importance of a balance of public and private interests when providing government support for the production of biosimilars. |  |
|  |  | Teran et al. (2022) | LAC |  | Regional manufacturing should be considered for small markets. Financial risk sharing through managed entry agreements or a subscription model could provide incentives for manufacturers to produce biosimilar medicines. |  |
|  |  | Zapatero Miguel (2015) | General |  | Technology transfer can support regional and national manufacturing in places with less experience. |  |
|  |  | Lee et al. (2016) | Northamerica | US | Perverse incentives by originator manufacturers to pay generic manufacturers for delay should be subject to antitrust laws. |  |
| **Market authorization (regulatory approval** | | |  |  |  |  |
| **1** | **Period of market exclusivity** | Vargas et al. (2022) | LAC |  | Data exclusivity of originator products prevents market entry of off patent medicines. Some Latin American countries with the strongest protections of intellectual property rights in the region are Chile, Colombia, Mexico, Peru, and those in Central America. |  |
|  |  | Lee et al. (2016) | Northamerica | USA | Incentives for generic manufacturers in offering the first generic manufacturer to file a market authorization within 180 days of market exclusivity. |  |
|  |  | Zapatero Miguel (2015) | General |  | Need to strengthen antitrust authorities to prevent abusive practices and increase competition. |  |
|  |  | Wouters et al. (2017) | North America | USA | Allow generic manufacturers to challenge patents without lengthy and costly litigation. Regulators should prevent pay-for-delay arrangements. |  |
| **2** | **No linkage between intellectual property and market authorization** | Dylst et al. (2015) | Europe | Italy | Abolish patent linkage |  |
|  |  | Dylst et al. (2013) | Europe |  | Marketing authorization procedures should remain independent of intellectual property protection considerations. |  |
| **3** | **Bolar exemption** | Vargas et al. (2022) | LAC |  | In Latin America and the Caribbean, it has been enacted by Brazil in 1996, Argentina in 1997, Colombia, Dominican Republic and Uruguay in 2000, Peru in 2009, and Chile in 2012. |  |
|  |  | Kaplan et al. (2012)* | General |  | The “Bolar” provisions, as an example of a TRIPS flexibility, could improve the availability of generics. |  |
|  |  | King & Kanavos (2002)* | Europe |  | The Bolar Rule allows generic firms to enter the market immediately after patent expiry. Without it, originator companies gain de facto patent extensions during the time needed for bioequivalence testing, delaying competition and potential cost savings. |  |
| **4** | **Regulatory reliance for biosimilar medicines** | PAHO (2022) | LAC |  | Reliance strategies support countries to navigate the complexities for regulating biosimilar medicines. The Caribbean Public Health Agency’s Caribbean Regulatory System participates in the WHO collaborative procedures for biosimilars and has also developed approval criteria, including that biosimilar medicines must be approved in selected reference authority markets. |  |
| **5** | **Clinical trial for biosimilars** | Dawkins Cox (2012) | LAC | Caribbean | Unlike chemical generic drugs, applications for approval of biosimilars cannot piggyback on the clinical data of their reference product. Manufacturers should provide clinical trial data for each submission made to regulatory agencies. These parameters are significant due to the potential for life-threatening complications that could arise. |  |
|  |  |  |  |  |  |  |
|  |  | Lizarraga & Mysler (2019) | LAC |  | Most Latin American countries (Brazil, Mexico, Argentina, Colombia, Chile, Paraguay and Peru) follow the WHO guidelines on biotherapeutic products or follow regulatory approval practices similar to those of the US Food and Drug Administration (FDA) or the European Medicines Agency (EMA). Bolivia and Ecuador are working on biosimilar regulations. |  |
|  |  | Scheinberg et al. (2018) | LAC | Brazil | Differences against the innovator biological product must be detected by independent phase III outcome studies. |  |
|  |  | PAHO (2022) | LAC |  | Clinical studies are required to assess comparability and safety. Although international regulation recommends that reference biotherapeutics are licensed with full data on quality, safety and efficacy in a given country, LAC national regulatory authorities differ in their requirement for reference biotherapeutics. |  |
|  |  | Olech (2016) | General |  | Rigorous early-stage structural, functional, and analytical testing, followed by nonclinical and clinical analyses comparing a biosimilar with its reference product, are required to demonstrate biosimilarity in regulatory markets worldwide. |  |
| **6** | **Bioequivalence (including biowaiver exceptions for generics)** | da Fonseca (2015) | LAC | Brazil | The paper focuses on the terminology of generic medicines in Latin America, highlighting the role of bioequivalence (BE) requirements, which had been implemented in Brazil and Mexico at the time. The adoption of BE was supported by generic manufacturers, who positioned their products as high-quality and equivalent to brand-name drugs. Pro-Genéricos emphasized BE as proof of safety and efficacy, linking it to broader public health goals and helping to build trust in generics. |  |
|  |  | Atal et al. (2023) | LAC | Chile | Quality regulation increased the demand for generic drugs by resolving asymmetric information and reducing aversion against unbranded generics, which induced the entry of high-quality medicines in place of low-quality drugs. Consumer welfare increased despite higher prices and a lower number of firms. |  |
|  |  | King & Kanavos (2002)* | Europe |  | Bioequivalence as part of regulatory approval and proof of quality as a pre-condition for all other policies promoting off-patent medicines. |  |
|  |  | Kaló et al. (2015)* | General |  | Common generic drug policies are registration-based on bioequivalence to the originator or reference. |  |
| **7** | **Fast-track approval for off-patent medicines** | Dias & Romano-Lieber (2006) | LAC | Brazil | Brazil introduced a “registro simplificado” (simplified registration) process for generic drugs, enabling faster approval by waiving requirements such as clinical trials—provided bioequivalence and other technical standards were met. This approach aimed to expand access to medicines by accelerating the availability of generics while maintaining their quality and efficacy. |  |
|  |  | Wouters et al. 2017) | United States and Europe |  | Report on the relevance of national regulators streamline the generic drug approval process. |  |
| **8** | **Market registration fees** | Kaplan et al. (2012)* | General |  | Policies that could improve the availability of generics include reduced fees for market authorization applications of generic medicines. |  |
|  |  | Wouters et al. (2017) | General |  | To address backlog of dossier appraisal by National Medicines Authorities, they would charge generic firms fees to increase their resources available for the drug approval process. |  |
| **Pricing & reimbursement** | | |  |  |  |  |
| **1** | **Price Regulation (price link)** | Kanavos (2014) | Europe | Several European countries | Evidence that linkage of prices of the generics to the originator medicine, such as in Greece, Italy, France, shows a significantly slower price reduction over time than countries that do not have this policy (UK, Germany, Denmark, the Netherlands). |  |
|  |  | Wouters et al. (2017) | North America and Europe | US and several European countries | The first generic on the market is priced at a certain percentage lower than the originator product. |  |
|  |  | Kaló et al. (2018)* | General |  | Mandatory price reduction to new generics linked to the reference product. |  |
|  |  | Río-Álvarez & Cruz-Martos (2024) | Europe | Spain | Although there is no specific rule in place, the first biosimilar medicine typically receives a 20-30% price reduction compared to the reference medicine. |  |
| **2** | **Reimbursement policies** | Bertoldi et al. (2019) | LAC | Brazil | Zero copayment for certain medicines can incentivize patients to fill their prescription. An increase in copayments and any additional payments for patients can result in lower prescription filling rates. |  |
|  |  | Dylst et al. (2013) | General |  | Tiered co-payment can incentivize patients to choose the lower price generic: the lowest co-payment for the generic and a higher co-payment for originator product. |  |
|  |  | Kanavos (2014) | Europe |  | The reimbursement price of the originator product is linked to the originator product. |  |
|  |  | Cameron et al. (2012) | General |  | Restricting reimbursement to the lower-cost products can reduce and stabilize retail prices. |  |
| **3** | **Mark-up regulation** | Leon et al. (2024) | LAC |  | Regulation of distribution mark-up which adds an aggregated 41% to the ex-factory market value in the region could result in significant price reductions in LAC. |  |
|  |  | Cameron et al. (2012) | General |  | Incentives for wholesalers and retailers to supply generic medicines, for example, regressive mark-up schemes that allow greater margins for lower-priced products. Fixed percentage margins can incentivize stocking and selling of more expensive including originator products. |  |
| **Procurement & supply chain** | | |  |  |  |  |
| **1** | **Procurement/Tender** | Kaplan et al. (2012)* | LMICs |  | Tendering should promote procurement of low price but quality assured products. |  |
|  |  | Moorkens et al. (2021) | Europe | Belgium | Hospital financing systems may incentivize the use of originator products. Enforcement of the practical implementation of public procurement law can promote the uptake of biosimilars. Tenders at regional and national level can promote economy of scale. Multiple winners should be selected to minimize supply shortages. Time of contracts should not be longer than two years to stimulate market dynamics. |  |
|  |  | Vogler et al. (2017) | Europe |  | Tendering is usually done by hospitals, or networks of hospitals, often with auction elements to enhance competition. Tenders need to be well designed and based on a sound framework to minimize the risk of no savings, litigation challenges or supply shortages. |  |
|  |  | Rosselli (2023) | LAC |  | Centralized procurement has resulted in savings for governments in several Latin American countries. |  |
|  |  | Kaló et al. (2018)* | General |  | Central tendering in public reimbursement |  |
|  |  | Puig-Junoy (2010) | Europe |  | Incentives to promote generic medicines include particular types of procurement contracts such as rebate contracts, portfolio contracts, collective preference policy, individual preference policy, index pricing and step price. |  |
|  |  | Wouters et al. (2017) | Europe |  | Tendering can lower administrative cost and reduce prices as well as increase price transparency. |  |
|  |  | Main et al. (2022) | Western Pacific | New Zealand | Annual bidding process for supply contracts of generic medicines. A contract for 2-3 years provides certainty for both the supplier and the buyer. Contractual arrangements are made to minimize risks. |  |
|  |  |  |  |  |  |  |
|  |  |  |  |  |  |  |
| **2** | **Supply chain support for generic medicines** | Zapatero Miguel (2015) | LMIC |  | Increasing the number of suppliers can increase competition. Pooled procurement can incentivize suppliers through volume guarantees. |  |
|  |  | Scheinberg et al. (2018) | LAC | Brazil | Involvement of the supplier of input materials in the partnerships to incentivize production of biosimilars is recommended. |  |
| **3** | **Regulation to make reference product available** | Lee et al. (2016) (generic medicines). | General |  | The need for regulation in place to facilitate access to reference branded products. |  |
| **Prescribing, dispensing and use** | | |  |  |  |  |
| **1** | **Advertisement and promotion regulations** | Alpert et al. (2023) | General |  | Advertising on originator products also had sizeable positive spillover effects on non-advertised generic drugs. |  |
| **2** | **Labeling of generics/biosimilars** | da Fonseca (2015) | LAC |  | In Latin American countries there is no regional regulatory standardization regarding the outer package labeling of brand-name or INN off patent products. |  |
|  |  | Stojanova et al. (2020) | LAC | Chile | In Chile, all bioequivalent medicines must be clearly marked with a yellow stripe and a bioequivalence certification seal. These products can be identified either as "generic bioequivalents," sold under their active ingredient name, or as "branded bioequivalents," marketed under a commercial brand name. |  |
|  |  | Dias & Romano-Lieber (2006) | LAC | Brazil | In Brazil, generic medicines are required to have a distinctive yellow stripe on their packaging—specifically Pantone 116C—which wraps around the main faces of the box. Within this stripe sits a prominent blue letter “G”, often accompanied by the words *“Medicamento Genérico – Lei 9.787/99”* in clear, uppercase lettering |  |
| **3** | **Pharmacovigilance (particularly for biosimilar medicines)** | Dawkins Cox (2012) | LAC | Caribbean | Pharmacovigilance measures should be implemented to monitor all biopharmaceuticals, particularly those originating from countries with less stringent regulatory measures. |  |
|  |  | Scheinberg et al. (2018) | LAC | Brazil | Post-marketing pharmacovigilance is crucial for detecting and monitoring rare or uncommon adverse events, as well as other issues related to efficacy. However, this process can be significantly hindered by the absence of a clearly defined naming system for biosimilars. |  |
|  |  | PAHO (2022) | LAC |  | Long-term safety needs to be ensured, which requires effective pharmacovigilance |  |
|  |  | Camacho (2017) | General |  | The need for standardization of regulatory requirements including the need for pharmacovigilance programs worldwide. |  |
|  |  | (Olech (2016) | North America and Europe | US and European countries | Report on the need for an effective pharmacovigilance program. |  |
| **Demand-side measures** | | | | | |  |
| **Prescriber focused policies** | | |  |  |  |  |
| **1** | **Prescribing by International Nonproprietary Names (INN)** | Tobar (2008) | LAC | Argentina | International nonproprietary name prescribing is a tool to increase competition and steer patients to the lower priced product. However, over time, prices may converge and lower the overall savings. |  |
|  |  | Kaló et al., (2018)* | General |  | International nonproprietary name prescribing. |  |
|  |  | Dylst et al. (2015) | Europe |  | Focus on a number of policies to stimulate the prescription of generic medicines such as prescription budgets, prescription quota, enforcement of INN prescribing, implementation of electronic prescribing, reduction of the use of the non-substitution clause |  |
|  |  | Swartenbroekx et al. (2014)* | Europe | Belgium, France, Germany, the Netherlands, Spain and Sweden | Biosimilar promotion through prescription quotas/target, clinical guidelines, primary substitution, reference price system, fixed payment and public tendering. |  |
| **2** | **Prescription guidelines & quotas** | Machado et al. (2024)* | Europe |  | Quotas for prescribing a certain percentage of biosimilar medicines, often accompanied by financial incentives and guidelines when substitution is permitted. “Gain sharing” as a model where savings are reinvested in healthcare for the benefit of all involved parties. |  |
|  |  | Dylst et al. (2013) | General |  | Physicians can be stimulated and supported to preferentially prescribe generic medicines through various mechanisms, including guidance, encouragement to prescribe by international non-proprietary name (INN), benchmarking, financial incentives, prescribing targets, and prescribing restrictions. |  |
|  |  | Moorkens et al. (2021) | Europe | Belgium | Good examples of biosimilar medicines where quotas are used in Europe to incentivize. |  |
|  |  | Lobo & Río-Álvarez (2021) | Europe | Spain | Incentives to include the use of biosimilar medicines could include sharing savings associated with more efficient use of medicines, at the same time, as any efficiencies made will be invested back into patient care to improve their health outcomes. Other incentives include making physicians eligible to receive financial incentives for certain biosimilar uptake. |  |
| **3** | **Interchangeability of biosimilars at the point of prescribing** | Teran et al. (2022) | LAC |  | Guidelines are necessary to advise on the interchangeability of biosimilars with the originator products. |  |
|  |  | PAHO (2022) | LAC |  | In 2022, there were no specific requirements on interchangeability. |  |
| **4** | **Education and awareness campaigns targeting prescribers** | Moorkens et al. (2021) | Europe | Belgium | The importance of raising awareness and education among healthcare providers, starting in medical school. Education activities should be run by independent organizations and use proven communication channels such as guidelines and letters. |  |
|  |  | Dylst et al. (2015) | Europe |  | Emphasis on demand size policies especially improved training of physician on prescribing and medicine selection |  |
| **Dispenser-focused policies** | | |  |  |  |  |
| **1** | **Substitution of generic medicines at the point of dispensing** | Wouters et al. (2017) | Europe and Northamerica |  | Mandatory or voluntary substitution is a cornerstone of policies promoting generic medicines in Europe. There are only a few countries where substitution is forbidden. In the United States, substitution laws differ from state to state. There has been strong resistance from originator manufacturers and some clinicians to substitution. |  |
|  |  | Bertoldi et al. (2019) | LAC | Brazil | Generic substitution under the Farmacia Popular program was allowed incentivizing pharmacies to dispense the lowest cost generic medicines. |  |
|  |  | Atal et al. (2023) | LAC | Chile | In Chile, pharmacists are only allowed to give a generic medicine instead of a brand-name one if the prescription uses the generic name and a certified bioequivalent is available. Even though efforts have been made to reduce the number of choices doctors have when prescribing, many still write prescriptions using brand names only, which makes it more difficult to use generic medicines. |  |
|  |  | Stojanova et al. (2020) | LAC | Chile | The user's consent is necessary to substitute, which requires that users have knowledge and a positive attitude toward substitution. |  |
|  |  | Kaló et al. (2018)* | General |  | Generic substitution by pharmacists. |  |
|  |  | Vogler et al. (2017) | Europe |  | Over time, more European countries have moved towards mandatory substitution. This is not the case for biosimilar substitution, which is not widely implemented. |  |
|  |  | Dylst et al. (2015) | Europe |  | Focus on demand side interventions such as making pharmacists’ remuneration independent of prices of medicines as well as setting substitution targets. |  |
| **2** | **Education and awareness campaigns targeting dispensers** | Aguilera et al. (2023) | LAC |  | Perception about generic medicines varied between studies from different LAC countries. While some physicians are overwhelmingly supportive of generic substitution and see generic medicines as therapeutic equivalents, other studies found physicians to be more reluctant. Pharmacists were found to have a higher trust in generic medicines than physicians. |  |
|  |  | Machado et al. (2024)* | Europe |  | Evidence on the use of educational programs to promote biosimilar medicines. |  |
| **Patient/Public-focused policies** | | |  |  |  |  |
| **1** | **Educational/Awareness initiatives - users of off-patented medicines** | Cazap et al. (2018) | General |  | Importance of educating patients about biosimilar medicines. |  |
|  |  | Sewell et al. (2012) | North America | USA | Generic medicines are often perceived as non-interchangeable, of lower quality, and potentially causing more harm. |  |
|  |  | Dylst et al., 2015 | Europe |  | Among other demand side policies emphasize the relevant of improving the perception of generic medicines among patients and providing financial stimulus for patients to accept generic substitution through the reimbursement policies that require patient to pay the differential cost between originator and generic product. |  |
|  |  | Río-Álvarez & Cruz-Martos (2024) | Europe | Spain | There is a knowledge gap regarding patients' and the general public's understanding of biosimilar medicines and their perceptions. |  |
|  |  | Aguilera et al. (2023) | LAC |  | Patients and the general public generally know that generic medicines are lower-priced than originator medicines, but also believe that their efficacy, quality, and safety are lower than those of originator products. Similarly, patients are generally concerned about the efficacy of biosimilar medicines. |  |
|  |  | Mallam et al. (2017) | General |  | Studies from Europe, USA and Japan show that the majority of patients know that generic medicines are of lower cost than originator medicines and agree to take them instead of originator medicines. |  |
|  |  | Torres Serna et al. (2018) | LAC | Colombia | Generic medicines are often less trusted, and users tend to doubt their effectiveness. One-fourth of the participants think that they are falsified. |  |

***Literature reviews identifying policies to promote off patent medicines

1. **LITERATURE GAP ON FACTORS INFLUENCING THE ADOPTION AND UPTAKE OF GENERIC AND BIOSIMILAR MEDICINES.**


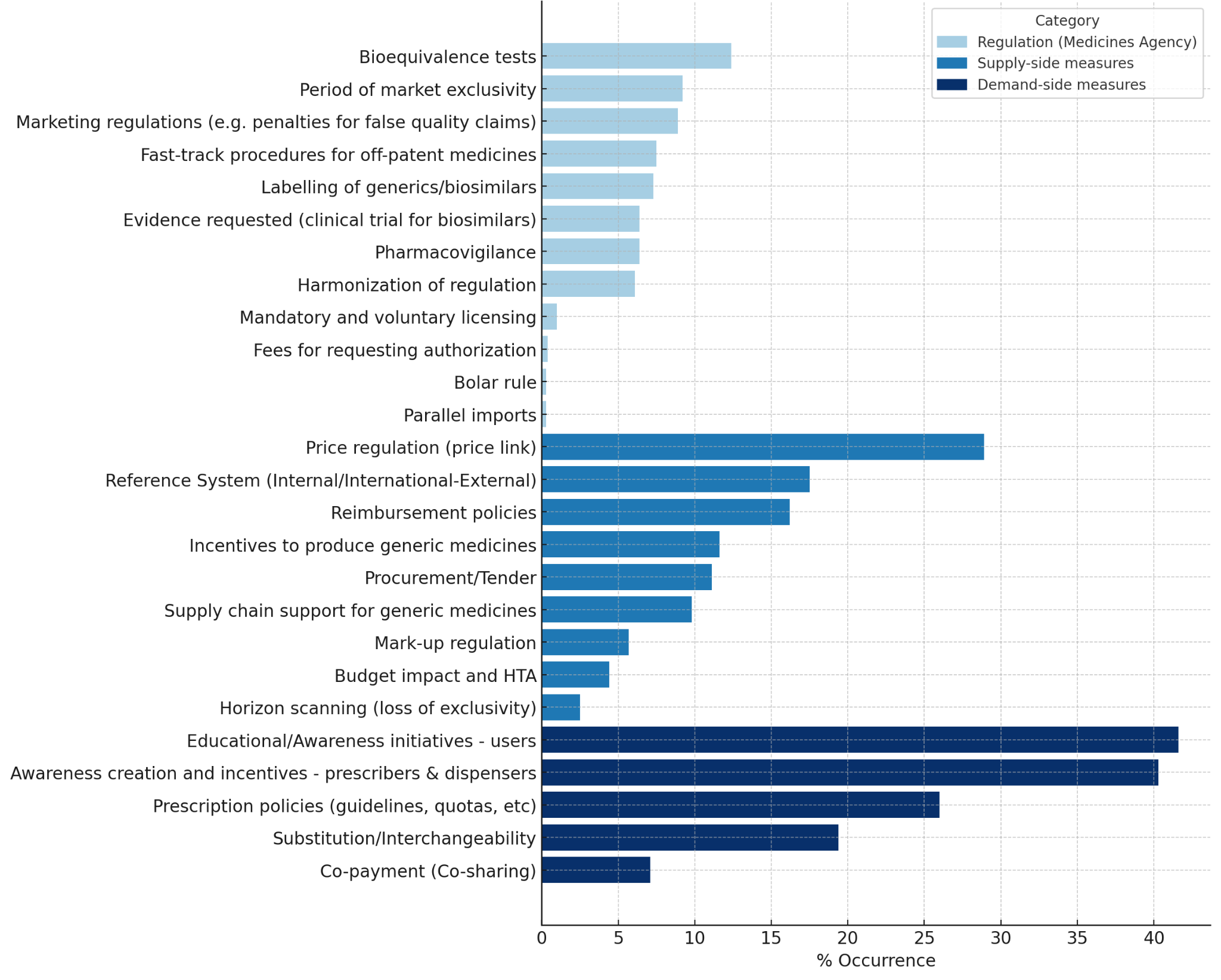


**Source**: Authors’ elaboration

1. **FRAMEWORK GLOSSARY**

**Supply-side policies**

| **Policy** | **Definition** |
| --- | --- |
| **R&D, innovation and manufacturing** | |
| Horizon screening | “The systematic identification of health technologies that are new, emerging or becoming obsolete and that have the potential to affect health, health services and/or society. An emerging health technology in this context is one that has not yet been adopted within the healthcare system. Pharmaceuticals are in the Phase II or III clinical trial, or pre-launch stage; medical devices are in the pre-marketing stage. A new health technology is one that is in the launch, early post-marketing, or early diffusion stages. Horizon scanning systems (e.g. early awareness and alert (EAA) systems) aim to support decision-making and the adoption and use of innovative technologies to the benefit of patients and health services^“^^[[1]](#footnote-1)^ |
| Incentives for local production | Measures designed to stimulate the domestic manufacturing of off-patent drugs through financial, regulatory, or institutional support. These may include tax exemptions, tax breaks, subsidies to domestic manufacturers to increase self-sufficiency, technology transfer agreements or infrastructure support. The aim is to strengthen local pharmaceutical capacity, improve medicine availability, and reduce reliance on imports. |
| **Market authorization (regulatory approval)** | |
| Period of market exclusivity | A period during which generic manufacturers cannot rely on the originator’s clinical trial data to obtain marketing approval for the same product? Something more?  [Trade Related Intellectual Property Rights (TRIPS) Article 33](https://www.wto.org/english/res_e/publications_e/ai17_e/trips_art33_jur.pdf) requires that patents be granted for a minimum term of 20 years from the filing date. It must be aligned with the proposed tool. |
| No linkage between intellectual property and market authorization | Drug regulators are tasked with assessing **safety, efficacy, and quality**, not enforcing patent rights. Linking IP to market authorization forces regulators to **monitor and enforce private patent claims**, which is outside their mandate and can lead to unnecessary bureaucratic delay. |
| Bolar exemption | Also known as “early working”, the “safe harbor” provision, or “regulatory review exemption”, the bolar exemption allows generic and biosimilar manufacturers to conduct research, testing, and regulatory submissions related to a patented medicine before the patent expires, without it being considered a patent infringement. This provision enables timely market entry upon patent expiration by reducing delays associated with regulatory approval processes.^[[2]](#footnote-2)^ |
| Clinical trials for biosimilars | Biosimilar medicines need to provide their own evidence from clinical trials. “Unlike chemical generic drugs, applications for approval of biosimilars cannot piggyback on the clinical data of their reference product. Manufacturers should provide clinical trial data for each submission made to regulatory agencies”. ^[[3]](#footnote-3)^ |
| Bioequivalence (including biowaiver exceptions for generics) | “Two medicinal products are bioequivalent if they are pharmaceutically equivalent or pharmaceutical alternatives and if their bio-availabilities after administration in the same molar dose are similar to such a degree that their effects, with respect to both efficacy and safety, will be essentially the same.” ^1^  Bioequivalence testing is used to determine whether the generic product is comparable with the originator product in terms of its therapeutic effect and safety profile. For some medicines, in vivo bioequivalence studies (which involve measuring the concentrations of the medicine in the bloodstream) are required. For others, regulatory authorities may grant a biowaiver, allowing in vitro testing, such as dissolution testing, to serve as a substitute for in-vivo studies. This approach is typically applied to drugs classified under the Biopharmaceutics Classification System (BCS), particularly BCS Class I (high solubility, high permeability) and sometimes Class III drugs. Dissolution testing evaluates the rate and extent to which a drug dissolves in the gastrointestinal environment, serving as a proxy for its absorption.  Quality Standards ensure that all pharmaceutical products—whether generic or innovator— meet stringent requirements for identity, strength, purity, and performance. These standards are defined by pharmacopeias (e.g., USP, EP) and enforced by regulatory agencies (e.g., FDA, EMA, WHO). Compliance with Good Manufacturing Practices (GMP) is also essential to maintaining product quality throughout production. |
| Fast-track approval for off-patent medicines | Also known as abbreviated or accelerated approval mechanisms. It refers to regulatory mechanisms that expedite the marketing authorization process for generic and biosimilar products, reducing approval times. Faster market entry leads to more competition with the goal of lowering prices. These pathways are designed to minimize administrative timelines and resource burdens while maintaining safety, efficacy, and quality standards. |
| Market registration fees | Market registration or market authorization fees refer to the charges levied by regulatory authorities for evaluating and granting marketing authorization for pharmaceutical products. Regulatory fees can serve as both an incentive and a barrier to the entry of biosimilars and generics, depending on their structure.^[[4]](#footnote-4)^  Lower authorization fees for generics than originator drugs may promote increased generic penetration rates. |
| **Pricing & reimbursement** | |
| Price regulation | It refers to policies and mechanisms used by regulatory authorities to control or influence the prices of pharmaceuticals. In the context of off-patent medicines, price regulation often includes setting maximum prices (i.e., price caps), applying internal reference pricing, mandating price reductions upon generic or biosimilar entry. These policies aim to ensure affordability, encourage competition, and contain public and out-of-pocket expenditure on pharmaceuticals. |
| Reimbursement policies | “Action by a government authority to decide whether the cost of a medicine is funded by a public payer (such as social health insurance or National Health Service), and if yes, to which extent and under which conditions.”^1^  Reimbursement policies play a crucial role in determining the accessibility and affordability of generic and biosimilar medicines. Countries use different reference systems to regulate reimbursement rates, ensuring cost-effectiveness. |
| Mark-up regulation | Mark-up regulations control the pricing structure at different levels of the supply chain, ensuring affordability.^[[5]](#footnote-5)^ This policy is designed to prevent excessive price inflation between manufacturers and patients, promote price transparency, and ensure the affordability of medicines, including generics and biosimilars. Mark-up controls may take the form of fixed margins, regressive mark-ups, or caps on profit margins, and are particularly relevant in contexts where out-of-pocket payments are high. |
| **Procurement & supply chain** | |
| Procurement/Tender | Procurement/tendering policies, particularly competitive tendering, have played a crucial role in reducing the cost of generic and biosimilar medicines. Monopolistic behavior, particularly strong for innovative medicines, can be in part compensated by centralized procurement^[[6]](#footnote-6)^. |
| Supply chain support for generic medicines | A well-functioning supply chain is critical for ensuring the availability of affordable generic and biosimilar medicines, particularly in resource-limited settings. Several approaches have been employed worldwide to enhance supply chain efficiency. For instance, centralized distribution networks have been adopted in some countries to improve availability and reduce the costs of generic medicines. |
| **Prescribing, dispensing and use** | |
| Labeling of generics/biosimilars | It refers to regulatory requirements that govern the information presented on the outer and inner packaging, and patient information. Labeling generic medicines that have demonstrated bioequivalence—whether branded or unbranded—in a way that clearly identifies them as off-patent or generic products is a policy tool used in several countries, including Brazil and Chile. This enhances transparency, builds public trust, and promotes the use of affordable alternatives, which is especially important in environments where patients may associate lower cost with lower quality and where related information is difficult to access.  For generics, labeling typically mirrors that of the originator product, with minor differences. For biosimilars, labeling may include additional data to reflect their nature as similar (but not identical) biological products, sometimes specifying the reference product used for comparison.  Clear, consistent labeling supports transparency, informed prescribing, pharmacovigilance, and public trust in the safety and efficacy of these medicines. |
| Pharmacovigilance (particularly for biosimilar medicines) | It is defined as science and activities relating to the detection, assessment, understanding, and prevention of adverse effects or any other drug-related problems. Its primary goal is to ensure the safety and efficacy of medicines throughout their lifecycle by monitoring and evaluating risks, particularly after market authorization.^[[7]](#footnote-7)^  Negative perceptions about generic and biosimilar medicines—such as being “inferior” to branded drugs—can be countered through transparent, well-functioning pharmacovigilance and post-market surveillance (PMS) systems. Pharmacovigilance allows for the monitoring of adverse drug reactions (ADRs) and therapeutic failures, which may reveal quality or formulation issues not apparent at the time of approval. Additionally, post-market surveillance helps identify substandard, degraded, or falsified products that may enter the market due to weak manufacturing practices, poor storage conditions, or counterfeit activity. |

**Demand-side policies**

| **Policy** | **Definition** |
| --- | --- |
| **Prescriber focused policies** | |
| Prescribing by International Nonproprietary Names (INN) | “Requirements for prescribers (e.g. physicians) to prescribe medicines by their INN, i.e. the active ingredient name instead of the brand name”^1^  This policy promotes the use of therapeutically equivalent alternatives, such as generics or biosimilars, and supports rational prescribing, cost containment, and medicine availability. INN prescribing can be mandated by regulation or encouraged through clinical guidelines and incentives. |
| Interchangeability of biosimilars at the point of prescribing | “An interchangeable pharmaceutical product is therapeutically equivalent to a comparator product and can be interchanged with the comparator in clinical practice.”^1^  This refers to a regulatory provision that allows a biosimilar medicine to be prescribed in place of its reference (originator) biological product without requiring prior approval from the prescriber. Interchangeability at this level relies on evidence demonstrating that the biosimilar produces the same clinical result as the reference product in any given patient^[[8]](#footnote-8)^. |
| Education and awareness campaigns targeting prescribers | It refers to structured interventions aimed at informing and influencing healthcare providers (particularly physicians) about the safety, efficacy, regulatory approval standards, and cost-effectiveness of generic and biosimilar medicines. These campaigns may include training sessions, clinical guidelines, dissemination of evidence, or decision-support tools shared with prescribers. The objective is to promote confidence in prescribing off-patent medicines, reduce misconceptions, and encourage rational and cost-conscious prescribing practices.  Public and healthcare professionals' awareness of generic and biosimilar medicines is crucial in improving their acceptance and utilization. Studies across different regions have highlighted various strategies for educating stakeholders and increasing trust in generics and biosimilars. Misconceptions about the quality and efficacy of generics persist, leading to lower adoption rates despite cost benefits. |
| **Dispenser-focused policies** | |
| Substitution of generic medicines at the point of dispensing | It refers to the practice of dispensing a generic version of a medication instead of the brand-name drug when a generic equivalent is available. |
| Education and awareness campaigns targeting dispensers | It refers to initiatives designed to inform and engage pharmacists and other medicine dispensers about the quality, safety, efficacy, and regulatory standards of generic and biosimilar medicines. These campaigns aim to build confidence in dispensing off-patent medicines, improve knowledge of substitution policies and pharmacovigilance responsibilities, and encourage the consistent implementation of cost-effective dispensing practices. Activities may include professional training, informational materials, continuing education, and integration into pharmacy curricula. |
| **Patient/Public-focused policies** | |
| Education and awareness campaigns targeting medicine users | It refers to interventions aimed at informing patients and the general public about the safety, effectiveness, and regulatory approval of generic and biosimilar medicines. These campaigns seek to address misconceptions, improve trust in off-patent products, and promote acceptance and adherence. Strategies may include mass media campaigns, community engagement, patient information leaflets, digital campaigns as well as collaboration with healthcare providers to deliver consistent messaging.  Public and healthcare professionals' awareness of generic and biosimilar medicines is crucial in improving their acceptance and utilization. Studies across different regions have highlighted various strategies to educate stakeholders and increase trust in generics and biosimilars. Misconceptions about the quality and efficacy of generics persist, leading to lower adoption rates despite cost benefits. Public trust in generics appears to be significantly higher in countries with well-structured information campaigns compared to those with minimal educational efforts. |

**References**

Aguilera, B., Peña, S., & Morales, J. P. (2023). Knowledge, Perceptions, and Utilization of Generics and Biosimilars in Latin America and the Caribbean: A Scoping Review. *Journal of Law, Medicine & Ethics*, *51*(S1), 100–115. https://doi.org/10.1017/jme.2023.117

Alpert, A., Lakdawalla, D., & Sood, N. (2023). Prescription drug advertising and drug utilization: The role of Medicare Part D. *Journal of Public Economics*, *221*, 104860. https://doi.org/10.1016/j.jpubeco.2023.104860

Atal, J. P., Zitko, P., Gutiérrez, C., & Giedion, Ú. (2023). *How Much Could Chilean Households  Save by  Using  Generics  Drugs  instead  of  their Branded  Equivalents? (and What would They Gain?)*. https://doi.org/10.18235/0005158

Bertoldi, A. D., Chaves, L. A., Ross-Degnan, D., Luiza, V. L., Emmerick, I. C. M., Silva, R. M. da, & Campos, M. R. (2019). Brazilian generics market change after Farmácia Popular program. *Revista de Saúde Pública*, *53*, 94. https://doi.org/10.11606/s1518-8787.2019053001237

Camacho, L. H. (2017). Current Status of Biosimilars in Oncology. *Drugs*, *77*(9), 985–997. https://doi.org/10.1007/s40265-017-0743-z

Cameron, A., Mantel-Teeuwisse, A. K., Leufkens, H. G. M., & Laing, R. O. (2012). Switching from Originator Brand Medicines to Generic Equivalents in Selected Developing Countries: How Much Could Be Saved? *Value in Health*, *15*(5), 664–673. <https://doi.org/10.1016/j.jval.2012.04.004>

Cazap, E., Jacobs, I., McBride, A., Popovian, R., & Sikora, K. (2018). Global Acceptance of Biosimilars: Importance of Regulatory Consistency, Education, and Trust. *The Oncologist*, *23*(10), 1188–1198. https://doi.org/10.1634/theoncologist.2017-0671

da Fonseca, E. (2015). *The Politics of Pharmaceutical Policy Reform: A Study of Generic Drug Regulation in Brazil*.

Dawkins Cox, S. (2012). Biosimilars in the Caribbean – Key Considerations. *West Indian Medical Journal*, *61*(9), 849–852. https://doi.org/10.7727/wimj.2012.331

Dias, C. R. C., & Romano-Lieber, N. S. (2006). Generic drug policy implementation in Brazil. *Cadernos de Saúde Pública*, *22*(8), 1661–1669. https://doi.org/10.1590/S0102-311X2006000800014

Dylst, P., Vulto, A., Godman, B., & Simoens, S. (2013). Generic Medicines: Solutions for a Sustainable Drug Market? *Applied Health Economics and Health Policy*, *11*(5), 437–443. https://doi.org/10.1007/s40258-013-0043-z

Dylst, P., Vulto, A., & Simoens, S. (2015). Analysis of the Italian generic medicines retail market: recommendations to enhance long-term sustainability. *Expert Review of Pharmacoeconomics & Outcomes Research*, *15*(1), 33–42. https://doi.org/10.1586/14737167.2014.950234

Kaló, Z., Holtorf, A. P., Alfonso-Cristancho, R., Shen, J., Ágh, T., Inotai, A., & Brixner, D. (2015). Need for Multicriteria Evaluation of Generic Drug Policies. *Value in Health*, *18*(2), 346–351. https://doi.org/10.1016/j.jval.2014.12.012

Kanavos, P. (2014). Measuring performance in off-patent drug markets: A methodological framework and empirical evidence from twelve EU Member States. *Health Policy*, *118*(2), 229–241. https://doi.org/10.1016/j.healthpol.2014.08.005

Kaplan, W. A., Ritz, L. S., Vitello, M., & Wirtz, V. J. (2012). Policies to promote use of generic medicines in low and middle income countries: A review of published literature, 2000–2010. *Health Policy*, *106*(3), 211–224. https://doi.org/10.1016/j.healthpol.2012.04.015

King, D. R., & Kanavos, P. (2002). Encouraging the use of generic medicines: implications for transition economies. *Croatian Medical Journal*, *43*(4), 462–469.

Lee, C.-Y., Chen, X., Romanelli, R. J., & Segal, J. B. (2016). Forces influencing generic drug development in the United States: a narrative review. *Journal of Pharmaceutical Policy and Practice*, *9*(1), 26. https://doi.org/10.1186/s40545-016-0079-1

Leon, G., Gonzalez-Pier, E., Kanavos, P., Ruiz de Castilla, E. M., & Machinicki, G. (2024). The 30-Billion-Dollar Distribution Markups and Taxes of Pharmaceuticals in Latin American Countries: Impact, Options, and Trade-Offs. *Value in Health Regional Issues*, *44*, 101015. https://doi.org/10.1016/j.vhri.2024.101015

Lizarraga, A., & Mysler, E. (2019). Similar and mimics: Latin America biosimilar regulations. *International Journal of Rheumatic Diseases*, *22*(1), 6–8. https://doi.org/10.1111/1756-185X.13461

Lobo, F., & Río-Álvarez, I. (2021). Barriers to Biosimilar Prescribing Incentives in the Context of Clinical Governance in Spain. *Pharmaceuticals*, *14*(3), 283. https://doi.org/10.3390/ph14030283

Machado, S., Cruz, A., Ferreira, P. L., Morais, C., & Pimenta, R. E. (2024). Policy measures and instruments used in European countries to increase biosimilar uptake: a systematic review. *Frontiers in Public Health*, *12*. https://doi.org/10.3389/fpubh.2024.1263472

Main, B., Csanadi, M., & Ozieranski, P. (2022). Pricing strategies, executive committee power and negotiation leverage in New Zealand’s containment of public spending on pharmaceuticals. *Health Economics, Policy and Law*, *17*(3), 348–365. https://doi.org/10.1017/S1744133122000068

Mallam, N., Byalakere Rudraiah, C. S., & Rudraswamy, S. (2017). Generic drugs: Current status and future potential. *Journal of Generic Medicines: The Business Journal for the Generic Medicines Sector*, *13*(2), 54–59. https://doi.org/10.1177/1741134317691803

Moorkens, E., Vulto, A. G., & Huys, I. (2021). Biosimilars in Belgium: a proposal for a more competitive market. *Acta Clinica Belgica*, *76*(6), 441–452. https://doi.org/10.1080/17843286.2020.1761690

Olech, E. (2016). Biosimilars: Rationale and current regulatory landscape. *Seminars in Arthritis and Rheumatism*, *45*(5), S1–S10. https://doi.org/10.1016/j.semarthrit.2016.01.001

PAHO. (2022). *Regulatory system strengthening in the americas lessons learned from the national regulatory authorities of regional reference*. https://iris.paho.org/handle/10665.2/53793

Pimenta, M. V., & Monteiro, G. (2019). The production of biopharmaceuticals in Brazil: current issues. *Brazilian Journal of Pharmaceutical Sciences*, *55*. https://doi.org/10.1590/s2175-97902019000217823

Puig-Junoy, J. (2010). Políticas de fomento de la competencia en precios en el mercado de genéricos: lecciones de la experiencia europea. *Gaceta Sanitaria*, *24*(3), 193–199. https://doi.org/10.1016/j.gaceta.2009.12.003

Río-Álvarez, I., & Cruz-Martos, E. (2024). Overview of biosimilar medicines in Spain: market dynamics, policies, evidence-based insights and avenues for a sustainable market. *Expert Opinion on Biological Therapy*, *24*(7), 583–597. https://doi.org/10.1080/14712598.2024.2363229

Rosselli, D. (2023). High cost drugs in Latin America: access and barriers. *Expert Review of Pharmacoeconomics & Outcomes Research*, *23*(6), 619–623. https://doi.org/10.1080/14737167.2023.2207825

Scheinberg, M. A., Felix, P. A. O., Kos, I. A., Andrade, M. D. A., & Azevedo, V. F. (2018). Partnership for productive development of biosimilar products: perspectives of access to biological products in the Brazilian market. *Einstein (São Paulo)*, *16*(3). https://doi.org/10.1590/s1679-45082018rw4175

Sewell, K., Andreae, S., Luke, E., & Safford, M. M. (2012). Perceptions of and Barriers to Use of Generic Medications in a Rural African American Population, Alabama, 2011. *Preventing Chronic Disease*, *9*, 120010. https://doi.org/10.5888/pcd9.120010

Stojanova, J., Lutz, M., Lazcano, G., & Arancibia, M. (2020). Chilean legislation on bioequivalence and biosimilarity and current controversies related to drug interchangeability. *Medwave*, *20*(01), e7825–e7825. https://doi.org/10.5867/medwave.2020.01.7825

Swartenbroekx, N., Farfan-Portet, Espín, J., & Gerkens, S. (2014). Incentives for market penetration of biosimilars in Belgium and in five European countries. *Journal de Pharmacie de Belgique*, (4), 36–46.

Teran, E., Gomez, H., Hannois, D., Lema, M., Mantilla, W., Rico-Restrepo, M., McElwee, E., Castro Sanchez, N., Valdivieso, N., & Espinoza, M. A. (2022). Streamlining breast cancer and colorectal cancer biosimilar regulations to improve treatment access in Latin America: an expert panel perspective. *The Lancet Oncology*, *23*(7), e348–e358. https://doi.org/10.1016/S1470-2045(22)00121-8

Tobar, F. (2008). Economía de los medicamentos genéricos en América Latina. *Revista Panamericana de Salud Pública*, *23*(1), 59–67. https://doi.org/10.1590/S1020-49892008000100008

Torres Serna, C., Salcedo, D. A., Fernández Rodríguez, L. M., Orozco Idrobo, L., & Rosero Alayón, R. A. (2018). Percepción de usuarios de Cali-Colombia acerca de medicamentos genéricos. *Revista Colombiana de Ciencias Químico-Farmacéuticas*, *47*(2), 151–168. https://doi.org/10.15446/rcciquifa.v47n2.73964

Vargas, V., Rama, M., & Singh, R. (2022). *Pharmaceuticals in Latin America and the Caribbean Players, access, and innovation across diverse models*. https://hdl.handle.net/10986/36871

Vogler, S., Gombocz, M., & Zimmermann, N. (2017). Tendering for off-patent outpatient medicines: lessons learned from experiences in Belgium, Denmark and the Netherlands. *Journal of Pharmaceutical Health Services Research*, *8*(3), 147–158. https://doi.org/10.1111/jphs.12180

Wouters, O. J., Kanavos, P. G., & McKe, M. (2017). Comparing Generic Drug Markets in Europe and the United States: Prices, Volumes, and Spending. *The Milbank Quarterly*, *95*(3), 554–601. https://doi.org/10.1111/1468-0009.12279

Zapatero Miguel, P. (2015). Legal and policy foundations for global generic competition: Promoting affordable drug pricing in developing societies. *Global Public Health*, *10*(8), 901–916. https://doi.org/10.1080/17441692.2015.1014824

1. WHO Collaborating Centre for Pharmaceutical Pricing and Reimbursement Policies (2025): Glossary of pharmaceutical terms. Vienna: Gesundheit Österreich (GÖG / Austrian National Public Health Institute). Available from: ppri.goeg.at/ppri-glossary. [↑](#footnote-ref-1)
2. Dawkins Cox, S. (2012). Biosimilars in the Caribbean – Key Considerations. West Indian Medical Journal, 61(9), 849–852. https://doi.org/10.7727/wimj.2012.331 [↑](#footnote-ref-2)
3. Wouters, O. J., Kanavos, P. G., & McKe, M. (2017). Comparing Generic Drug Markets in Europe and the United States: Prices, Volumes, and Spending. The Milbank Quarterly, 95(3), 554–601. https://doi.org/10.1111/1468-0009.12279. [↑](#footnote-ref-3)
4. Wouters, O. J., Kanavos, P. G., & McKe, M. (2017). Comparing Generic Drug Markets in Europe and the United States: Prices, Volumes, and Spending. The Milbank Quarterly, 95(3), 554–601. https://doi.org/10.1111/1468-0009.12279. [↑](#footnote-ref-4)
5. Vogler, S., Gombocz, M., & Zimmermann, N. (2017). Tendering for off-patent outpatient medicines: lessons learned from experiences in Belgium, Denmark and the Netherlands. Journal of Pharmaceutical Health Services Research, 8(3), 147–158. https://doi.org/10.1111/jphs.12180 [↑](#footnote-ref-5)
6. European Commission et al. (2022). *Study on best practices in the public procurement of medicines: final report*. Luxembourg: Publications Office of the European Union. https://data.europa.eu/doi/10.2925/044781. [↑](#footnote-ref-6)
7. World Health Organization. The Importance of Pharmacovigilance: Safety Monitoring of Medicinal Products [Internet]. Geneva: WHO; 2002. Available from: <https://apps.who.int/iris/handle/10665/42493> [↑](#footnote-ref-7)
8. European Medicines Agency and Heads of Medicines Agencies. (2023). Statement on the scientific rationale supporting interchangeability of biosimilar medicines in the EU. Available at: <https://www.ema.europa.eu/en/documents/public-statement/statement-scientific-rationale-supporting-interchangeability-biosimilar-medicines-eu_en.pdf>. [↑](#footnote-ref-8)
